# What Is the Best Vancomycin Therapeutic Drug Monitoring Parameter to Assess Efficacy? A Critical Review of Experimental Data and Assessment of the Need for Individual Patient Minimum Inhibitory Concentration Value

**DOI:** 10.3390/microorganisms11030567

**Published:** 2023-02-24

**Authors:** Bruce R. Dalton

**Affiliations:** Pharmacy Department, Alberta Health Services, Calgary, AB T2N 2T9, Canada; bruce.dalton@ahs.ca

**Keywords:** vancomycin, pharmacokinetics, pharmacodynamics, therapeutic drug monitoring

## Abstract

Therapeutic drug monitoring is recommended for the use of vancomycin, but a recent widely publicized US medical society consensus statement has changed the suggested optimal method(s) of dose adjustment. Specifically, 24 h area under the curve (AUC_24_)-based monitoring is has been recommended for vancomycin in preference to monitoring of trough concentrations. One reason cited for this change is the claim that AUC_24_ is a superior correlate to efficacy than trough (Cmin). Evidence from a number of retrospective analyses have been critically reviewed and determined to have weaknesses. This narrative review focuses on the experimental studies performed in vivo in animal models of infection and in vitro to determine the extent to which these data may provide a compelling distinction between pharmacokinetic/pharmacodynamics (PKPD) parameters that may translate to clinical use in therapeutic drug monitoring. Animal in vivo studies have been presented at conferences, but no original peer reviewed studies could be found that compare various PKPD parameters. These conference proceeding findings were supportive but unconvincing, even though they were favorably presented subsequently in review articles and clinical practice guidelines. In vitro data are somewhat conflicting, but the range of concentrations may play a role in the discrepancies found. It has been suggested that MIC may be assumed to have a value of 1 mg/L; however, it can be demonstrated that this assumption may lead to considerable discrepancy from results with an actual MIC value. The AUC_24_ parameter has been weighed against the percentage of time above the MIC (%T > MIC) as a comparative PKPD parameter, yet this may be an inappropriate comparison for vancomycin since all clinically useful dosing provides 100% T > MIC. Regardless, there is a distinction between clinical TDM parameters and PKPD parameters, so, in practice, the change to AUC_24_:MIC based on animal experiments and in vitro evidence for vancomycin may be premature.

## 1. Introduction

Therapeutic drug monitoring (TDM) is the clinical practice of adjusting dose based on quantitative assessment(s) of a drug from individual patient tissue or fluid samples. Vancomycin is a commonly used intravenous antibiotic for Gram positive bacteria, and it demonstrates considerable inter-individual pharmacokinetic characteristics and toxicity (nephrotoxicity, ototoxicity, dermatologic reactions, and phlebitis) within the therapeutic range, requiring routine dose assessment and adjustment using blood concentration determination to ensure efficacy and avoid toxicity [1,2,3].

A recently updated consensus statement, “Therapeutic monitoring of vancomycin for serious methicillin-resistant Staphylococcus aureus infections: A revised consensus guideline and review by the American Society of Health-System Pharmacists, the Infectious Diseases Society of America, the Pediatric Infectious Diseases Society, and the Society of Infectious Diseases Pharmacists”, published in multiple journals and endorsed by multiple practice societies, suggests clinicians should perform TDM based on 24 h area under the concentration–time curve to minimum inhibitory concentration, assuming an MIC value of 1 mg/L, (AUC_24_:MIC) for serious methicillin-resistant *Staphylococcus aureus* (MRSA) infections, in preference to trough (Cmin) TDM [2,4,5,6]. Subsequent to the updated consensus statement, two other groups have published clinical practice guidelines reiterating the recommendation to target an AUC:MIC > 400 for efficacy (assuming MIC = 1) [7,8], but one also comments that an alternative consideration is trough monitoring with the range of 10–20 mg/L [8]. Clinical investigations of TDM parameters supporting this change of practice were largely retrospective assessments of the AUC_24_:MIC at one time point in therapy in patients who actually had TDM management of their vancomycin dosing using trough assessment, a critical study design flaw, which, unfortunately, was repeated many times [9,10,11,12,13,14,15,16,17,18,19]. While the updated consensus review is a valiant effort to condense an enormous amount of study data, the recommendations have been reviewed and criticized for other important study shortcomings as well [20,21,22]. One of the key drivers of the recommendation to perform TDM using AUC:MIC (assuming an MIC value = 1 mg/L) in preference to trough TDM was the perception or theoretical conception that AUC:MIC is a superior pharmacokinetic/pharmacodynamic (PKPD) index that correlates better with efficacy compared to other PKPD parameters [1,23]. 

There are three distinct PKPD parameters traditionally used to determine the action of an antibiotic at various concentrations, which may have implications for dosing regimens. These are: time of drug concentration in serum above the minimum inhibitory concentration measured as a percentage of the dosing interval (%T > MIC); maximum or peak concentration (Cmax); and area under the concentration time curve (AUC). Seminal reviews in the early years of PKPD and animal model research have not discussed therapeutic drug monitoring as a primary or even a potential secondary purpose of this subscience [24,25,26]. Table 1 highlights the differences between PKPD and TDM parameters. While Cmax and AUC_24_ are directly equivalent to the above PKPD parameters, use of %T > MIC has been avoided in vancomycin TDM in favor of trough concentration, likely due to the impractical nature of obtaining the MIC. The value of TDM using any candidate parameter should be shown in clinical study(s) that dose adjustment using the candidate parameter results in improved relevant patient outcomes. This has not been the case for TDM of vancomycin using any of the candidate parameters (AUC_24_, AUC_24_:MIC, or trough) [1,2,18]. Furthermore, there may not be any clinically important differences between TDM parameters for vancomycin, as correlation between trough and AUC_24_ in published clinical studies has been found to be generally strong [19], and a high degree of covariance between PKPD variables is expected [27]. PKPD parameters from non-human animals cannot be presumed to be equivalent in humans. Mice are noted to have much faster vancomycin elimination, with half-lives under an hour; therefore, the relationship of trough to AUC may be very different in small animals compared to humans [28,29,30]. 

The objective of this review is to appraise the preclinical PKPD evidence supporting the widely held belief that the AUC_24_:MIC of vancomycin is the parameter that has strongest correlation to efficacy, particularly in MRSA, and to re-examine available study data using novel methods to determine the impact of the assumption of an MIC value of 1 mg/L. 

## 2. Materials and Methods

Searches in PubMed and Google Scholar for pre-clinical original research were conducted using terms “vancomycin”, “pharmacodynamics”, “area under the curve” or “AUC” and “time over minimum inhibitory concentration”, “time above minimum inhibitory concentration”, or “T > MIC”, without restrictions on date or languages. Bibliographies of review articles and guidelines were examined for all possibly relevant papers. We excluded studies solely focused on bacteria other than *Staphylococcus aureus*, including methicillin sensitive *Staphylococcus aureus*, due to relative lack of MRSA-specific data. 

Citation trails were followed from guidelines and published review articles to source reports of original research. 

Data from publications were thoroughly interrogated and charts were reconstructed if bias in graphical presentation was possible. 

Because of the confusion between the first vancomycin consensus statement [1] and the update [2] in their recommendations for the use of an actual MIC value (AUC:MIC) versus the assumption of an MIC value of 1 mg/L (i.e., numerically equivalent to AUC), and the fact that many of the clinical studies assessing the association of efficacy to AUC were conducted using AUC:MIC with an actual MIC value by broth microdilution, a simple data simulation experiment was conducted. Monte Carlo simulations using an available sample of second-day vancomycin AUC_24_ data from a recent publication [31] and random assignment of an MIC value from a worldwide surveillance project of >77,000 clinical MRSA isolates [32] was conducted to assess the expected difference between AUC_24_ and AUC_24_:MIC. AUC_24_ data were extracted from a graph in the former publication using an online plot digitizing application (https://apps.automeris.io/wpd/ (accessed on 1 October 2022)) and randomly assigned an MIC value following a distribution of values of the latter study. A total of 520,000 sample MCS were conducted. Correlation was assessed by square of the Pearson correlation coefficient (coefficient of determination) and bias was assessed with the linear regression coefficient. Calculations and graph construction were conducted with Microsoft Excel, and are available in Supplementary Materials.

## 3. Results

### 3.1. Paucity of Peer Reviewed Published Studies from In Vivo PKPD Studies

A lengthy discussion of PKPD parameters as they relate to vancomycin was included in the first version of the Consensus Review of American Society of Health Systems Pharmacy and the Infectious Diseases Society of America [1], which stated: “investigators have concluded that the AUC/MIC is the pharmacodynamically linked parameter for measuring vancomycin’s effectiveness”, citing two review papers [23,25]. These two reviews discussed two non-peer reviewed experiment reports in neutropenic mouse thigh model infections from conferences in 1987 and 1999, as well as some in vitro experiments. The abstracts of the neutropenic mouse thigh reports are available in perpetuity, but full details of results and methods are not [29,31]. 

**Figure 1:**
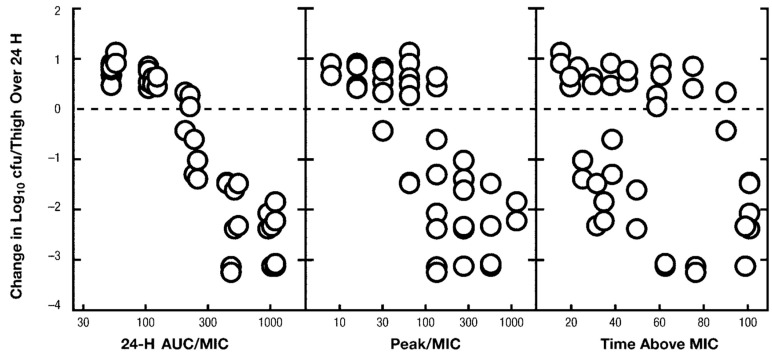
Depiction of the results of the methicillin sensitive *Staphylococcus aureus* (1 strain, MIC = 1 mg/L) experiment by Ebert et al., 1987 [29] from the review article by Rybak MJ, 2006 (ref. [23], reproduced with permission). Three pharmacokinetic/pharmacodynamics indices are plotted against 24 h change in bacterial count in thighs of mice. Note the methicillin-resistant strain results were not presented nor discussed in the text of the review.

In the first of these studies, based on the abstract by Ebert et al. [29] and review by Rybak, MJ [23], 24 h bacterial count change as a function of area under the curve (AUC, note: not AUC: MIC), peak level to MIC (Cmax), or time above the minimum inhibitory concentration (%T > MIC) in methicillin susceptible *Staphylococcus aureus* (MSSA) and MRSA (1 strain of each, MIC = 1 mg/L for each) were compared [29]. In the review by Rybak, MJ, the MSSA data were presented in graphic form as AUC:MIC, but, oddly, the MRSA data were not included in this graph, nor was there an explanation of the omission or an explanation of the change in the PKPD parameter notation from AUC to AUC:MIC [23]. From the abstract, log peak level (Cmax) and log AUC were found to be predictors of efficacy versus MSSA, while only log AUC was found to be a good predictor for MRSA. Statistical analysis of correlation was not provided, but the graphic representation (Figure 1) of the data qualitatively appears to support the author’s claims. 

**Figure 2:**
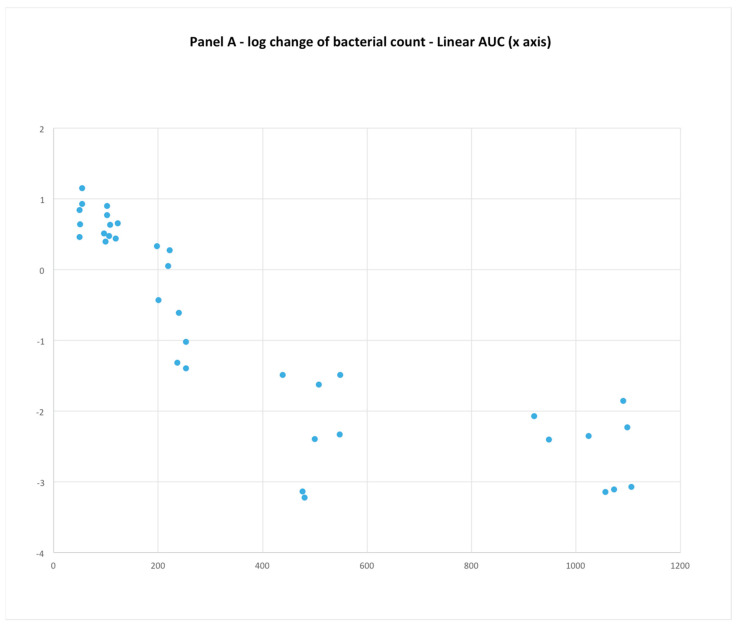

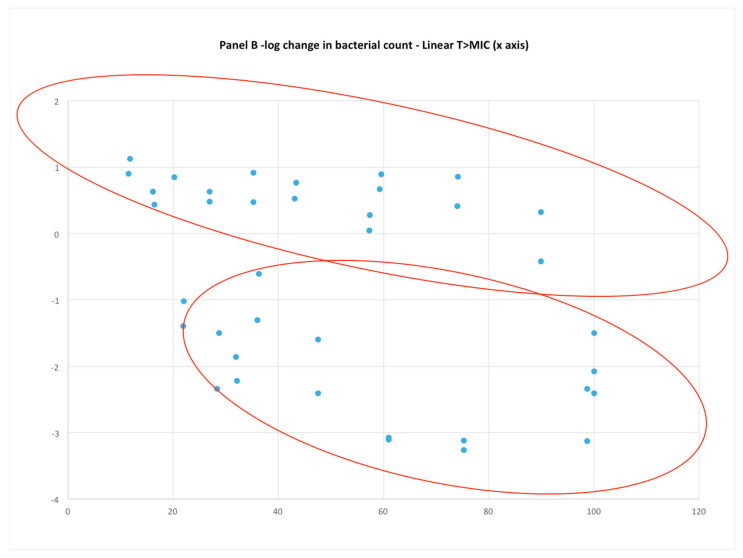
Panel **A**: Data from Figure 1 ([23]), first panel replotted on graph with linear scale x−axis. Correlation evident but visual assessment of scatter is less hidden by the compression on a log scale. Panel **B**: Data from Figure 1, third panel replotted on similar graph size and axes scale to Panel **A** for comparison purposes. Interesting heterogeneity in response indicated by red colored loops.

The first panel (AUC) clearly appears to have the strongest relationship of dependent to independent variable (Figure 1). However, the range of the AUC values presented in log scale are greater than %T > MIC (linear scale). Since the correlation was only assumed due to visual interpretation, and since the use of the log scale on a graph’s axis can compress space, making the correlation appear stronger, data from both plots were extracted using a plot digitizer program (https://apps.automeris.io/wpd/ (accessed on 1 October 2022)) and replotted on graphs with linear x-axes (Figure 2) in order to equalize the comparisons. While it is still clear that the AUC has a stronger relationship to change in bacterial count, visually, the scatter of data points in both graphs seems considerable. There is also a notable heterogeneity of response in the %T > MIC data, but, without a complete published report of the methods and results, it is very difficult to speculate on why these findings were observed. 

The second experiment, presented at a 1999 conference, determined AUC/MIC, %T > MIC and Cmax:MIC with vancomycin intermediate (VISA, *n* = 2) and vancomycin susceptible (VSSA, *n* = 3) strains [33]. The authors reported that %T > MIC was not a predictor of efficacy, without details of the data, but that the AUC:MIC and Cmax:MIC did significantly predict efficacy as assessed by AUC:MIC or Cmax:MIC at 50% maximum efficacy compared to those receiving no therapy. However, the results are confusing because the VISA strains (MIC = 8) responded at lower AUC:MIC and Cmax:MIC compared to the VSSA strains (MIC = 0.5–1). Given the small scale of this experiment, lack of peer review, and the contradictory results, it is difficult to deem this report as reliable evidence. 

In addition to these conference reports, peer reviewed mouse thigh infection model study reports determined that AUC:MIC of vancomycin were found, but there are no comparisons to other PKPD parameters [30,34]. 

### 3.2. Is Evidence from In Vitro Models Consistent with In Vivo Studies?

Several investigators have published time–kill studies using in vitro pharmacokinetic systems that observed bacterial count change in broth at time points over 12–24 h but without direct measurement of PKPD parameters (Table 2) [35,36,37,38,39,40]. 

**Figure 3:**
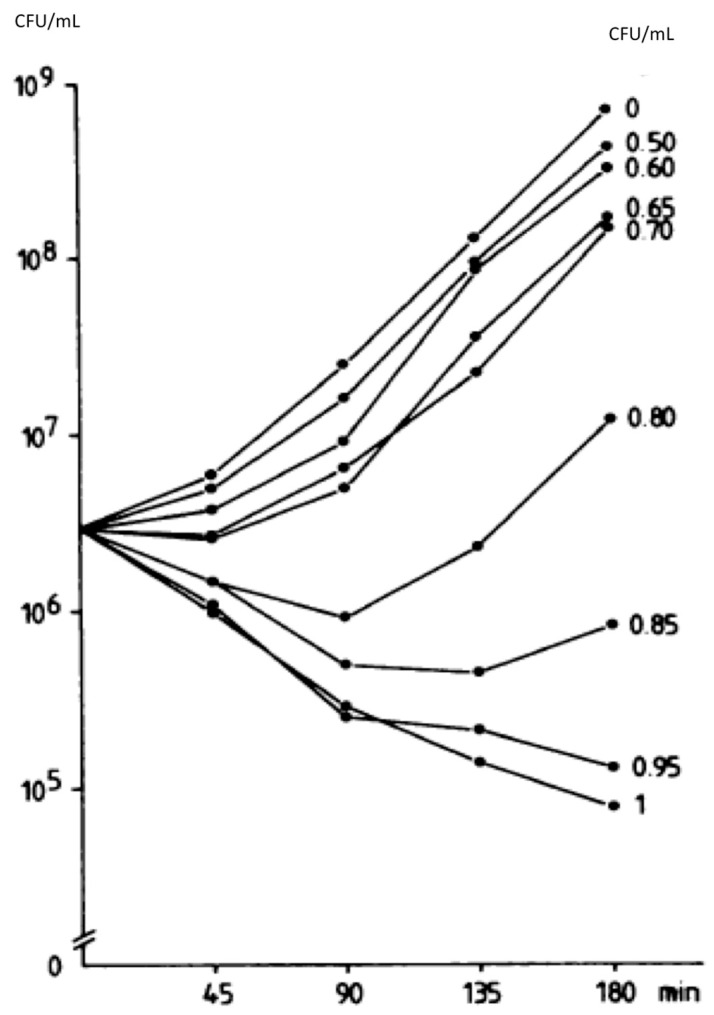
From Peetermans et al. ([40], reproduced with permission) Number of CFU of *Staphylococcus aureus* in vitro in the presence of various concentrations of vancomycin during 3 h exposure.

Results from these experiments consistently failed to find a relationship of bacterial killing to the drug concentrations tested, and they led some to conclude that %T > MIC is the most relevant PKPD parameter [39]. However, it is important to consider that the complete lack of concentration dependence is an untenable concept. That is, if one reduces the dose or concentration of vancomycin from very high to very low, there will be a point at which it is ineffective or less effective. This implies that consideration of the range tested is crucial. The above cited studies roughly simulated concentrations used in clinical practice (see Table 1). However, Peetermans et al. [40] also used concentrations of 0.5 to 1 mg/L and found a clear concentration to response effect; results using concentrations above 1 mg/L were not displayed, but were, in text, described. They stated that there was no greater killing above 1 mg/L [40] (Figure 3). 

**Figure 4:**
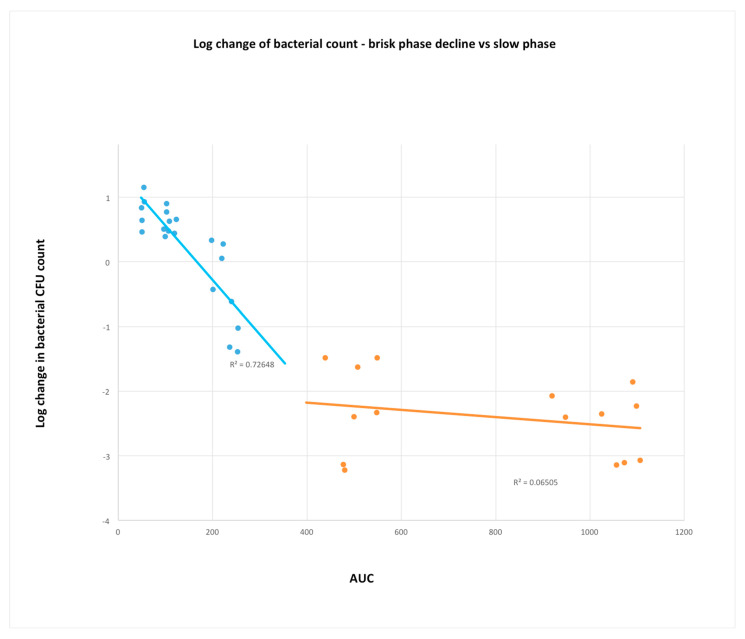
MSSA data from Ebert et al. ([29]) extracted from graphs of Rybak 2006 ([23]). Area under the curve versus log change in bacteria over 24 h in neutropenic mouse−thigh infection model. Replotted with linear *x*−axis and divided dataset by responsiveness. Blue datapoints and regression line represent a brisk response at AUC ~50–400. Orange datapoints and regression line demonstrate AUC values where very little concentration-dependent activity is observable.

How can these data in vitro data be reconciled with the findings of Ebert et al. [23,29]? It is evident from Figure 1, panel on the left, that the greatest change in CFU count accompanies the AUC range from ~50 to 400 mg·h/L, above which there is little greater effect in bacterial count change (Figure 4). Based on these limited data, there appears to be a levelling off of concentration dependence above AUC ~250 and below ~420. Therefore, concentration dependence and advantage of AUC over trough in terms of correlation to bacterial kill in animal models is mostly from what would be considered clinically suboptimal dosing. It is noteworthy that the clinical retrospective studies used to support AUC:MIC monitoring have determined widely ranging values (from 211 to 660) as the threshold for clinical efficacy [18,19]. 

Keeping in mind that AUC, the measure of exposure to a drug, is the product of concentration and time, but time, the width of the “area”, is a fixed constant at a standard time value (in this case 24 h). Therefore, comparison of different AUC values is simply a comparison of average concentration values (multiplied by a constant: 24 h). Division of AUC by 24 will give the average concentration, so to compare the AUCs of ~50–420 in the brisk phase of response (Figure 1), where concentration dependence is observed, is approximately equal to an average concentration of 2–16 mg/L over the 24 h experimental period. In vitro concentrations would be expected to be more potent than those in in vivo use due to penetration and protein binding in serum; therefore, one might expect concentration-dependent killing, consistent with the results observed by Peetermans et al. [40], providing evidence of the use of AUC or Cmax as a PKPD parameter correlating with efficacy at concentrations that would generally be regarded as considerably subtherapeutic. 

Therefore, if AUC or AUC:MIC is the PKPD parameter of choice due to correlation to efficacy, it is based upon experimentation at subtherapeutic or borderline therapeutic concentrations. In vitro data demonstrating concentration independence above a certain threshold support %T > MIC as a PKPD parameter within the therapeutic concentrations of vancomycin, although AUC_24_, as a measure of average concentration, is unlikely to differ significantly in utility from trough since, experimentally, concentration dependence has not been observed within the clinically therapeutic range. 

### 3.3. Is the Use of MIC Required in PKPD Parameter Based TDM?

Available evidence, above, could support AUC_24_:MIC as one PKPD parameter for TDM of vancomycin. The guideline recommendation of the updated consensus statement suggests clinicians assume an MIC = 1, or, effectively, use the AUC_24_ value only as a practical measure [2]. Ebert et al. [29] calculated AUC but did not utilize strains of different MICs, while others calculated AUC:MIC using actual MIC [30,33,34]. While one might conclude the early data of Ebert et al. would support the use of AUC_24_ alone, it did not test the influence of variation in MIC value to AUC:MIC of vancomycin against *Staphylococcus aureus*, and it would seem premature to form conclusions based on non peer reviewed findings presented at a conference greater than 30 years ago. 

An actual MIC by broth microdilution (the gold standard), or other non-automated methods, is not usually available to clinicians at the start of therapy, and MIC estimates by automated susceptibility systems have been shown to produce considerably different results compared to broth microdilution or e-test methods commonly used in retrospective cohort studies [41,42]. It is increasingly recognized that there are considerable limitations associated with use of MIC as the measure of in vitro potency of an antibiotic, which may, rightly, apply to TDM of vancomycin [43,44]. While the guideline recommendation to forgo use of MIC in TDM (i.e., assumption of a value of 1 mg/L) is convenient, it invalidates the supporting preclinical and clinical evidence that did calculate the PKPD parameter with broth microdilution MIC as the denominator, creating a conundrum for guideline authors and TDM practitioners alike. Furthermore, it has not been clearly communicated if PKPD evidence supports or refutes the use AUC without MIC [2]. 

### 3.4. Monte Carlo Simulations of AUC_24_ and AUC_24_:MIC (Methods Described above)

**Figure 5:**
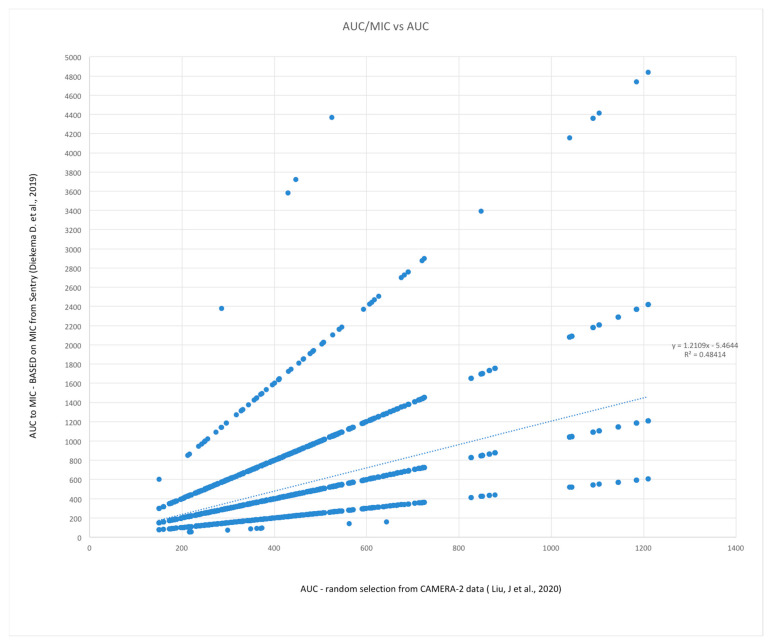
Example of one of five (n = 20,000) Monte Carlo simulations—random pairing of AUC_24_ (*X*−axis, values from [31]) to an MIC value to calculate expected AUC_24_:MIC (*Y*−axis, values from [32]). Correlation in this simulation R^2^ = 0.484. β = 1.211 See text for details.

MCS experiments are data simulations that can be used to predict how variables may randomly combine, based on the distribution variables values in previous studies. A total of 5 MCS were conducted using a sample size of 20,000 pairing an AUC_24_ value with a randomly assigned MIC from a worldwide surveillance project following the distribution of variable values reported in two large studies [31,32]. Simulation results are presented in Table 3, and a graphical example of results of one simulation is presented in Figure 5. Table 4 demonstrates the agreement and discrepancy rates expected with the two forms of dosing adequacy assessment. Overall agreement occurred in 81.1% of assessments, a value similar to the proportion with an MIC = 1 (74.3%) in the surveillance project [32]. AUC_24_:MIC values are distributed along lines associated with discrete MIC values, but, overall, the coefficient of determination (R^2^) was approximately 50% (R^2^ range 0.484–0.508) for each simulation, and bias was near 20% (coefficient range 1.170–1.210). The MCS clearly show that the AUC would not be expected to be an accurate representation of AUC_24_:MIC in the real world. Given that MIC = 0.5 is half the value of MIC = 1.0, and the second most common individual MIC value in the referenced surveillance project (20.5%) [32], one could reasonably expect that dosing to achieve a minimum AUC_24_:MIC of 400 mg.hr/L would be 50%, as compared to dosing to achieve an AUC of 400, and thus providing substantially lower exposure to the toxic effects of vancomycin.

Programing and complete results are available in Supplementary Materials.

### 3.5. Does %T > MIC Represent the Activity of troughs of Vancomycin in the Clinical Setting?

For concentration independent inhibiting antibiotics such as beta-lactams, %T > MIC has been a PKPD parameter of interest, showing a sigmoidal correlation of %T > MIC to the change in bacterial counts in infection models [25]. However, vancomycin has been dosed in clinical practice to achieve troughs many-fold greater than the MIC of susceptible staphylococcal strains. In other words, in clinical scenarios, vancomycin %T > MIC is essentially always 100% of the dosing interval, yet data from Rybak MJ [23], originating from the Ebert et al. experiment [29] in the mouse thigh, demonstrate various levels of response in lower than therapeutic concentrations, which is of dubious relevance to TDM (Figure 1). PKPD experiments examining the correlation of T > 2xMIC, T > 5XMIC, or T > 10XMIC related to vancomycin could not be found, but would be of great interest for vancomycin [45]. 

## 4. Conclusions

The development of animal models of infection has been important for the understanding of antibiotic mechanisms and the optimization of dosing strategies. This work led to the development of concepts such as time- and concentration-dependent killing and PKPD parameters which best represent the action of the drugs. In the case of vancomycin, reports of in vivo experimental work comparing the relationship of different PKPD parameters to efficacy have not been peer reviewed. The available peer reviewed literature that can be used to evaluate different PKPD parameters in vitro is limited and somewhat inconsistent, and these models do not allow direct calculation of PKPD parameters. The recommendation to assume an MIC value = 1 mg/L is convenient but would be expected to underestimate true AUC_24_:MIC and only account for 50% of variability in the true AUC_24_:MIC value, and, therefore, belies the nature of precision dosing. The assumption, that the “best” PKPD parameter in experiments derived using various ranges of clinically relevant and irrelevant concentrations is applicable to the clinical setting of TDM, is unsupported. It may be that any PDPK parameter is reasonable for use in TDM because of an inherent correlation of these measures. Therefore, TDM of vancomycin using AUC_24_ or trough may be reasonable until it becomes clear from clinical and/or experimental data that one parameter is superior for the purpose of TDM. While equipoise still exists, health care institutions should perform TDM using the most convenient and cost-efficient parameter.

## Figures and Tables

**Table 1:** Characteristics of pharmacokinetic/pharmacodynamic parameters versus therapeutic drug monitoring parameters.

	Pharmacokinetic/Pharmacodynamic (PKPD) Parameter	Therapeutic Drug Monitoring (TDM) Parameter
Purpose	Understanding of drug mechanism, empiric dosing for general therapeutic target	Adjusting dose to individualized target
Setting	Experimental model	Clinical
Use of MIC	Use of MIC–reference method(s)	Delay to obtain MIC, usually from automated susceptibility method(s). In the case of vancomycin; assumed to equal 1 mg/L
Range of dosing	Very wide (orders of magnitude)	Human therapeutic range
Relevant outcomes	Bacterial kill Animal survival Organ damage/toxicity markers	Clinical cure Patient survival Toxicity
Specific examples	Time above the minimum inhibitory concentration (%T > MIC) Maximum or peak concentration (Cmax) to MIC ratio Area under concentration time curve (AUC, various time intervals) to MIC ratio	Trough or minimum concentration (Cmin) Cmax Twenty-four hour AUC (AUC_24_)

**Table 2:** In vitro pharmacodynamic studies of vancomycin.

Citation/Ref Number	Model	Vancomycin Concentrations (mg/L)	Results
Ackerman B et al., 1992 [35]	Static concentration— 24 h observation. *Staphylococcal* spp.	10, 20, 30 and 50	Statistically and visually no difference in bacterial kill in *Staphylococcus aureus*.
Cantoni L et al., 1990 [36]	Static concentration— 48 h observation *Staphylococcus aureus*	2, 10, 40	Visually slightly less activity of 2 mg/L vs. 10 and 40. No statistical test result reported.
Duffull S et al., 1994 [37]	Dynamic in vitro model—24 h observation *Staphylococcus aureus*	48→3 (one dose) 30→7.5 (two doses) 16.2 mg/L constant	No difference in rate or extent of bacterial killing.
Larsson A et al., 1996 [38]	Dynamic in vitro—12 h observation *Staphylococcus aureus* T1/2 = 6 h	5, 10, 20 or 40 mg/L peak concentration	5 and 20 mg/L slightly lower extent of killing than 10 and 40 mg/L by visual inspection. Statistically NS.
Lowdin E et al., 1998 [39]	Dynamic in vitro—24 h observation *Staphylococcus aureus* and *Staphylococcus epidermidis*. T1/2 = 5 h	2, 4, 8, 16 and 64× MIC	No concentration-dependent killing observed by visual inspection.
Peetermans W et al., 1990 [40]	Static concentrations—3 h observation, *Staphylococcus aureus*	0.5, 0.6, 0.65, 0.7, 0.8, 0.85, 0.95 and 1	Progressive effect on bacterial killing by increasing concentrations. Higher than 1 mg/L did not lead to more killing.

**Table 3:** Results of Monte Carlo Simulations—random pairing of AUC_24_ to MIC to calculate AUC_24_:MIC (see text for details).

Simulation Number	R-Square (Pearson, Correlation)	Bias (Linear Regression Coefficient)
1	0.498	1.198
2	0.508	1.210
3	0.500	1.202
4	0.492	1.170
5	0.484	1.211

**Table 4:** Agreement of dosing assessments by AUC versus AUC_24_:MIC number, (percentage of total) of Monte Carlo Simulation—see text for details.

	AUC:MIC High	AUC:MIC Therapeutic	AUC:MIC Low
AUC High	3171 (15.9)	44 (0.2)	120 (0.6)
AUC Therapeutic	1430 (7.2)	5312 (26.6)	359 (1.8)
AUC Low	994 (5.0)	825 (4.1)	7745 (38.7)

## Data Availability

Monte Carlo Simulation calculation, compiling and results are provided in Supplementary Materials.

## Supplementary Materials

The following supporting information can be downloaded at: https://www.mdpi.com/article/10.3390/microorganisms11030567/s1, Monte Carlo Simulation Excel file 1.1.
